# A Comparison of Deterministic and Stochastic Modeling Approaches for Biochemical Reaction Systems: On Fixed Points, Means, and Modes

**DOI:** 10.3389/fgene.2016.00157

**Published:** 2016-08-31

**Authors:** Sayuri K. Hahl, Andreas Kremling

**Affiliations:** Specialty Division for Systems Biotechnology, Faculty of Mechanical Engineering, Technische Universität MünchenGarching, Germany

**Keywords:** ordinary differential equations, chemical master equation, bistability, bimodality, gene expression, protein bursts

## Abstract

In the mathematical modeling of biochemical reactions, a convenient standard approach is to use ordinary differential equations (ODEs) that follow the law of mass action. However, this deterministic ansatz is based on simplifications; in particular, it neglects noise, which is inherent to biological processes. In contrast, the stochasticity of reactions is captured in detail by the discrete chemical master equation (CME). Therefore, the CME is frequently applied to mesoscopic systems, where copy numbers of involved components are small and random fluctuations are thus significant. Here, we compare those two common modeling approaches, aiming at identifying parallels and discrepancies between deterministic variables and possible stochastic counterparts like the mean or modes of the state space probability distribution. To that end, a mathematically flexible reaction scheme of autoregulatory gene expression is translated into the corresponding ODE and CME formulations. We show that in the thermodynamic limit, deterministic stable fixed points usually correspond well to the modes in the stationary probability distribution. However, this connection might be disrupted in small systems. The discrepancies are characterized and systematically traced back to the magnitude of the stoichiometric coefficients and to the presence of nonlinear reactions. These factors are found to synergistically promote large and highly asymmetric fluctuations. As a consequence, bistable but unimodal, and monostable but bimodal systems can emerge. This clearly challenges the role of ODE modeling in the description of cellular signaling and regulation, where some of the involved components usually occur in low copy numbers. Nevertheless, systems whose bimodality originates from deterministic bistability are found to sustain a more robust separation of the two states compared to bimodal, but monostable systems. In regulatory circuits that require precise coordination, ODE modeling is thus still expected to provide relevant indications on the underlying dynamics.

## 1. Introduction

In the last decades, the potential of mathematical modeling for the analysis of biological systems has widely been recognized. However, the reliability and explanatory power of such models depend greatly on the chosen modeling approaches, which may largely differ in several aspects like their level of detail or the approximations they are based on. This fact has led to debates not only with critics from other scientific fields, but also within the community of systems biologists (Gunawardena, 2014). Nowadays, modeling is still lacking any kind of gold standard, since it is highly specific toward the underlying systems biological problem. In fact, each and every model can only provide a rough depiction of nature, and a major challenge consists in applying or even developing modeling techniques which answer the questions to be addressed as best as possible with reasonable effort.

The host of existing modeling approaches can be grouped according to various criteria. One important classification distinguishes between deterministic and stochastic models. In deterministic modeling, stochasticity within the system is neglected. One of the most frequently used deterministic approaches consists in ordinary differential equations (ODEs), which are based on the phenomenological law of mass action. They provide a dynamic and quantitative description of spatially homogenous systems. Since ODEs are intensely used in other scientific fields as well, numerous analysis techniques and simulation methods have been developed thus far. In theoretical biology, ODEs have been applied to a wide range of problems, for example to the description of metabolism (Kremling et al., 2007), signaling (Shinar et al., 2007) or gene regulation within cells (Tyson and Othmer, 1978), to the investigation of systemic effects in complex multicellular organisms (Gallenberger et al., 2012), and to the analysis of population dynamics (Edelstein-Keshet, 1988).

However, biological systems are always subject to stochastic effects, which occur on all levels—from molecular to macroscopic. These can be captured by stochastic models. Concerning biochemical networks, the chemical master equation (CME) is very frequently applied (van Kampen, 2007, Chapter 5). Unfortunately, its analytical solution is usually intractable, especially if a large number of reactants is involved. The Gillespie algorithm provides exact simulations of trajectories of the master equation (Gillespie, 1977), but the computational cost is high for multi-component reaction systems. Therefore, several approximate variants of the CME as well as of the Gillespie algorithm have been developed (Gillespie, 2001; Chatterjee et al., 2005; Anderson, 2008).

Randomness plays a major role in signaling and regulation, where the copy number of the involved components is small and noise in gene expression is significant. Therefore, they are major application fields for stochastic models in systems biology (Tsimring, 2014). Compared to their deterministic counterparts, stochastic models are in general more difficult to analyze. Therefore, the need for incorporating stochasticity should be carefully elucidated, depending on the biological application.

In the following, we aim at comparing the explanatory power of the very detailed discrete CMEs and the corresponding ODEs. Starting from reviewing the analogies in their formulation in Section 2, we will then collect the parallels and discrepancies between the modeling results in Section 3. In this context, common concepts like bistability and bimodality will be contrasted. Unlike a couple of other studies on this topic, we will also regard mesoscopic systems which are not close to the thermodynamic limit. We will discuss these aspects in the context of a simple gene regulatory system, using it as a platform for identifying general factors which influence the comparability between these kinds of deterministic and stochastic models.

## 2. Theoretical background

### 2.1. Foundations of CMEs and ODEs

In this section, we will review the formulation of CMEs and ODEs for chemical reaction systems, and highlight the connection between deterministic reaction rates and stochastic reaction propensities. More detailed descriptions can be found, e.g., in Gillespie (2007) and van Kampen (2007).

#### 2.1.1. Chemical reactions as a Markov process and the chemical master equation

Let us consider a system containing molecules of *M* different chemical species (components) that can in total undergo *R* different irreversible, elementary reactions. These reactions can be of zeroth order (e.g., entry of molecules into an open system), of first order (e.g., degradation of compounds or unimolecular conversion) or of higher order (e.g., dimerization). In the latter case, random encounters of two or more molecules are necessary for the reaction to occur. The *j*-th reaction can be written as

(1) $$\sum_{i\;=\;1}^{M}\beta_{ij}\cdot X_{i}\;\to \;\sum_{i\;=\;1}^{M}\gamma_{ij}\cdot X_{i},$$

with *X*_*i*_ denoting the components in the system and β_*ij*_, $\gamma_{ij}\in {\mathbb{N}}_{0}^{+}$ being the stoichiometric coefficients of the educts and products. Assuming that the system is spatially homogeneous, its state can be characterized by the copy numbers of the components it contains. It can therefore be formulated in terms of a vector $\text{n}\left(t\right)={\left(n_{1}\left(t\right),\ldots ,n_{M}\left(t\right)\right)}^{⊤}$, where *n*_*i*_ denotes the copy number of the *i*-th component, and *t* is the time variable.

In the CME framework, the system state is modeled as a continuous-time stochastic process, for which the Markov property holds. This means that the probability distribution of future system states only depends on the present state, but not on past states (memorylessness). Here, we regard the discrete state space defined above. Let *p*_**n**_(*t*) be the probability of being in state **n** at time *t* and π(**n**, **m**) be the probability per infinitesimal time unit (propensity) of a transition from **m** to **n**. The CME is a reformulation of the Chapman-Kolmogorov equation and can be written as:

(2) $${\dot{p}}_{n}(t)=\sum_{m}(\pi (n,m)p_{m}(t)-\pi (m,n)p_{n}(t)),$$

where ṗ_**n**_(*t*) denotes the time derivative of the probability and the summation runs over the whole state space. The CME thus states that the temporal evolution of *p*_**n**_ is determined by the balance between transitions leading to state **n** and transitions away from **n**. Since Equation (2) applies to all states **n**, it defines a system of differential equations describing the dynamics of the probability mass function *p*.

Next, π(., .) needs to be defined in the context of the reaction system (Equation 1). Within infinitesimal intervals, transitions occur solely due to single reactions. The stoichiometric matrix **A** with entries *a*_*ij*_: = γ_*ij*_ − β_*ij*_ and columns ${\text{a}}_{j}={\left(a_{1j},\ldots ,a_{Mj}\right)}^{⊤}$ captures all possible transitions between states, so that the CME can be re-formulated as

(3) $${\dot{p}}_{n}(t)=\sum_{j\;=\;1}^{R}(w_{j}(n-a_{j})\cdot p_{n-a_{j}}(t)-w_{j}(n)\cdot p_{n}(t)).$$

Here, *w*_*j*_(**n**) is the propensity of the *j*-th reaction, which is the probability per infinitesimal time unit for the *j*-th reaction to occur, when the system is in state **n**. The propensities can more specifically be formulated as

(4) $$w_{j}(n)=\kappa_{j}\cdot \prod_{i\;=\;1}^{M}\left(\begin{array}{c} n_{i}\\ \beta_{ij} \end{array}\right).$$

Here, κ_*j*_ denotes the stochastic reaction constant, which is determined by physical properties of the reaction (e.g., activation energy, complexity) and by environmental conditions like temperature. The latter product reflects the combinatorial probability of random encounters of the educts: it accounts for reactive collisions of the components, where β_*ij*_ out of *n*_*i*_ molecules of the *i*-th component are involved.

#### 2.1.2. Formulation of a system of ordinary differential equations

We consider ODEs based on the law of mass action, which has originally been formulated by Guldberg and Waage. In this deterministic approach, concentrations instead of molecule numbers are usually regarded (Gillespie, 1976), and the state space is treated as continuous (Gillespie, 1977). This is only justified if the molecule numbers of the species and the system size *V* are sufficiently large. Let $c_{i}:=\frac{n_{i}}{V}$ be the concentration of the *i*-th reaction component. For constant *V*, the concentration change of the *i*-th component through the reactions in Equation (1) is given by

(5) $${\dot{c}}_{i}=\sum_{j\;=\;1}^{R}\left((\gamma_{ij}-\beta_{ij})\cdot k_{j}\cdot \prod_{l\;=\;1}^{M}c_{l}^{\beta_{lj}}\right)=\sum_{j\;=\;1}^{R}\left(a_{ij}\cdot k_{j}\cdot \prod_{l\;=\;1}^{M}c_{l}^{\beta_{lj}}\right).$$

*k*_*j*_ is the deterministic rate constant. The law of mass action thus states that the speed of a reaction depends on this constant and on powers of the concentrations of the educts. In case of elementary reactions, the exponents are determined by the stoichiometry of the reaction.

#### 2.1.3. Relation between stochastic and deterministic reaction constants

While the stochastic reaction constant reflects the likelihood of a reaction to occur, the deterministic counterpart is mostly interpreted as a kinetic term. However, the following mathematical relation holds:

(6) $$\kappa_{j}=k_{j}\cdot V\cdot \prod_{i\;=\;1}^{M}\frac{\beta_{ij}!}{V^{\beta_{ij}}}.$$

This equation is a generalized form of the relation derived in Gillespie (1977). The stochastic rate constant thus depends on the system size *V*, and this dependence is determined by the stoichiometry: While zero-order reactions are more likely to happen in large systems, the chance of molecular collisions required for higher-order reactions is reduced when the density of molecules decreases due to an expansion of *V*. Inserting the relation into Equation (4) yields

(7) $$w_{j}(n)=\frac{k_{j}}{V^{\sum_{i=1}^{M}\beta_{ij}-1}}\prod_{i\;=\;1}^{M}\frac{n_{i}!}{(n_{i}-\beta_{ij})!}.$$

For small and well-characterized chemical reaction systems, the CME and ODE formulations are straightforward. However, many biological reactions in cellular systems are complex and a description in terms of elementary reactions like in Equation (1) might thus be difficult. For example, the conversion of a protein with the help of an enzyme is actually a multi-step process. The description of gene expression including the actions of the transcription and translation machinery and under the influence of certain activators or repressors would be infeasible at this level of detail. By exploiting time scale separation, it is a general practice to lump several fast reactions into rate functions *k*_*j*_(**n**), which replace the constants *k*_*j*_ and which depend on the current system state (pseudo-steady-state assumption). They can for example be chosen to describe Hill-type kinetics.

### 2.2. The mean of the CME and its relation to the ODE system

The deterministic formulation is sometimes regarded as a description of average values, which are assumed to represent the system quite well when the molecule numbers of the components and the system size are large. However, a basic calculation of the mean of the CME shows that this analogy only holds true in special cases (cf. for example van Kampen, 2007, Chapter 5):

Let *N*_*i*_ be the stochastic variable of the copy number of the *i*-th component and let $\text{N}={\left(N_{1},\ldots ,N_{M}\right)}^{⊤}$. Let furthermore 𝔼[.] denote the expected value. Then, the ODE of 𝔼[*N*_*i*_] satisfies:

(8) $$\dot{𝔼}[N_{i}]=\sum_{n\in {\mathbb{Z}}^{M}}n_{i}{\dot{p}}_{n}=\sum_{j\;=\;1}^{R}\left(a_{ij}\cdot 𝔼[w_{j}(N)]\right).$$

For the sake of simplicity, we have omitted the time variable *t*. The derivation can be found in the Supplementary Material 1. Inserting the explicit formulation of the propensities (Equation 7) leads to

(9) $$\dot{𝔼}[N_{i}]=\sum_{j=1}^{R}\left(a_{ij}\cdot 𝔼\left[\frac{k_{j}}{V^{\sum_{i=1}^{M}\beta_{ij}-1}}\prod_{l=1}^{M}\frac{N_{l}!}{(N_{l}-\beta_{lj})!}\right]\right).$$

If all β_*lj*_ ≤ 1, or if the system is close to the thermodynamic limit (i.e., the theoretical limit *V* → ∞, *n*_*l*_ → ∞ s.t. *c*_*l*_ is constant), the approximation $\frac{n_{l}!}{\left(n_{l}-\beta_{lj}\right)!V^{\beta_{lj}}}\approx \frac{n_{l}^{\beta_{lj}}}{V^{\beta_{lj}}}=c_{l}^{\beta_{lj}}$ holds. The expectation of the random variable $C_{i}:=\frac{N_{i}}{V}$ describing the concentration then reads:

(10) $$\dot{𝔼}[C_{i}]=\sum_{j\;=\;1}^{R}\left(a_{ij}\cdot 𝔼\left[k_{j}\prod_{l\;=\;1}^{M}C_{l}{}^{\beta_{lj}}\right]\right).$$

In general, 𝔼[*f*(*Y*)] ≠ *f*(𝔼[*Y*]) for any nonlinear function *f*, where *Y* is an arbitrary random variable. A comparison between the ODE in Equation (10) and the deterministic formulation in Equation (5) thus shows that the deterministic variable *c*_*i*_ is only an exact description of 𝔼[*C*_*i*_], if the term $k_{j}\prod_{l=1}^{M}C_{l}^{\beta_{lj}}$ is linear. This holds true for zero and first order reactions with constant *k*_*j*_, which is quite a severe restriction in the context of biochemical processes.

### 2.3. Bistability vs. bimodality

In addition to the calculations in the preceding section, one further, quite obvious limitation in identifying deterministic variables with the mean of stochastic variables becomes apparent when multimodal probability distributions are regarded. They have more than one local maximum, each of them representing a characteristic composition of state variables that is “favored” by the system. Multimodality therefore reflects system state heterogeneity. This heterogeneity might be temporal (frequent switching of individual systems between different states) or population-based (split of a population into subgroups with different, but stable characteristics). If deterministic models were a mere description of the mean, they would obscure this multimodal structure and would therefore be rather uninformative. Indeed, a property of ODE models exists which describes some sort of heterogeneity: This property is called multistability, meaning that multiple stable fixed points can be assumed by the system. The initial condition determines which of the states the system will finally tend to. Although the effect of stochasticity, which might allow for random transitions between the stable states, is neglected, multistability has long been regarded as the deterministic equivalent of multimodality.

Recently, several theoretical as well as experimental studies have challenged this association. Bistable systems with a unimodal distribution have been observed as well as bimodal systems whose deterministic description predicted monostability (Artyomov et al., 2007; Qian et al., 2009; Bishop and Qian, 2010; Ochab-Marcinek and Tabaka, 2010; To and Maheshri, 2010; Shu et al., 2011; McSweeney and Popovic, 2014). We can thus conclude that deterministic variables are neither fully equivalent to the stochastic mean nor to stochastic modes. This raises the question under which conditions deterministic models can provide reliable information on system dynamics and which qualitative and quantitative conclusions can be drawn from the results.

In Gillespie (2007, 2009), Kurtz (1972, 1980), and van Kampen (2007), connections between deterministic and stochastic variables have been derived which are valid in the thermodynamic limit under certain constraints on the reaction system. These constraints are usually fulfilled for elementary reactions, but might be violated when multiple reactions are lumped together. Furthermore, in gene expression and regulation, where the molecule copy numbers of some of the involved components are low, the thermodynamic limit is not an appropriate approximation.

In order to characterize possible differences between ODE and CME models in a mesoscopic regime, we regard a flexible biochemical regulatory system that can be bimodal, depending on the parameters. Usually, bimodality arises due to positive feedback loops—a topological structure which can be found in autostimulatory gene expression systems, both natural and synthetic. They offer a fruitful platform for studying the effect of stochasticity in a biological context: Protein production was found to occur in bursts of random size, which enables us to study the influence of stochasticity and stoichiometry by varying the burst characteristics, namely the average amplitude and frequency. Moreover, by theoretically varying the feedback structure, the effect of nonlinear reaction rates can be studied. Using this system, we will determine in which aspects and to what extent the deterministic description is consistent with the CME. More general statements will then be derived from our observations.

## 3. Results

### 3.1. Modeling of a gene regulatory system with feedback

Our basic description of gene regulation is mainly adapted from Friedman et al. (2006) and Aquino et al. (2012). Instead of modeling the dynamics on the promoter, mRNA, and protein level in detail, we will use a simplification proposed in Aquino et al. (2012), which regards only protein formation and degradation. It is based on a time scale separation with subsequent averaging of promoter states and of mRNA concentrations. A discussion on the validity of this approximation is also given in Aquino et al. (2012). We will not put special emphasis on the accuracy of the model from a biological point of view, but rather on the mathematical characteristics of the model equations. We thus prefer the reduced model due to its analytical solvability.

The reaction scheme we consider is given by:

(11) $$\begin{array}{l} \emptyset \overset{\;\frac{1}{\mu^{*}}\;f(n)\;}{\to }\mu X\\ X\;\overset{\;\delta \;}{\to }\;\emptyset \end{array}$$

Here, *X* denotes the protein, which is generated in a burst with random size μ ∈ ℕ^+^. The burst size follows a geometric distribution with mean μ^*^. The rate of protein production is given by *f* : ℝ → ℝ, which is a smooth monotonically increasing function evaluated at the integer protein molecule number *n*, illustrating autostimulation. It is scaled by μ^*^ in order to obtain comparable results when the parameter is modified in our analyses (i.e., a change of the burst size is balanced by a reciprocal change of the burst frequency so that the mean protein level remains constant). The protein degradation rate is linear with parameter δ. Throughout this study, the cell volume *V* is assumed to be fixed, so that dilution effects are neglected.

This scheme is suitable for studying the effect of linear as well as of nonlinear functions *f*, by which different types of autoregulation can be represented. For example, a non-cooperative stimulatory effect of the protein on its own expression can be described by a linearly increasing function or by a Michaelis-Menten-type saturation function. Cooperative feedback, where several protein molecules exert a synergistic autoregulatory effect, can be described through a sigmoid Hill-type function. Furthermore, by choosing large values for μ^*^, significant jumps in the protein trajectories can be generated.

### 3.2. Deterministic description in terms of ordinary differential equations

Since stochasticity is neglected in deterministic descriptions, the number of proteins produced in each burst is assumed to be equal to the average burst size μ^*^. Let $c:=\frac{n}{V}$ be the protein concentration, which is treated as a continuous variable. The ODE is then given by:

(12) $$\dot{c}=\mu^{*}\frac{\frac{1}{\mu^{*}}\tilde{f}(c)}{V}-\delta c=\frac{\tilde{f}(c)}{V}-\delta c,$$

where $\tilde{f}(c):\text{​​}=f(c\;\cdot \;V)\;\forall c\in \mathbb{R}$. The steady state condition reads

(13) $$\frac{\tilde{f}(c)}{\delta }=cV.$$

**Figure 1:**
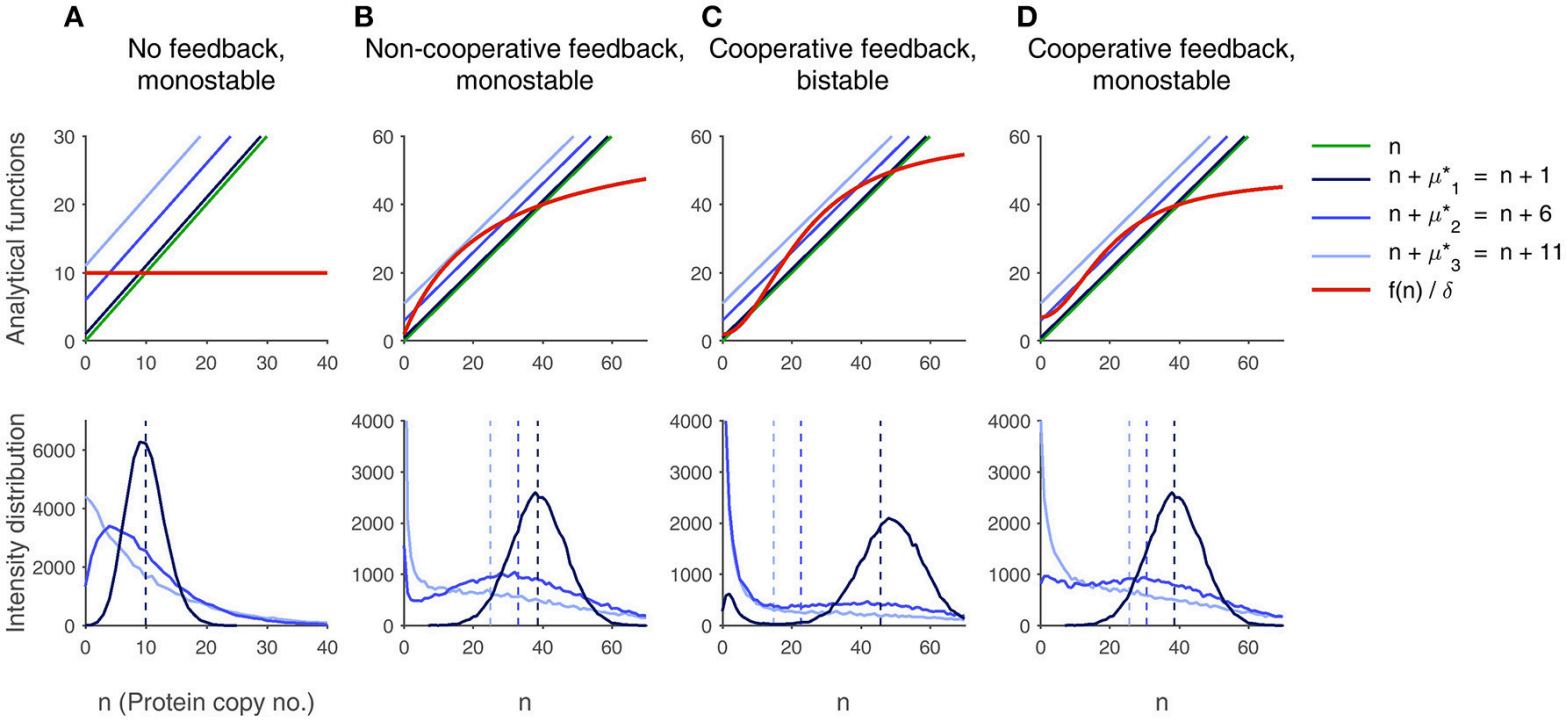
**Influence of bursting and of nonlinear feedback on the protein distribution**. From left to right, the feedback characteristics are varied. The top row shows the analytical results. The deterministic fixed points can be read from the value of *n* at the intersection of $\frac{f\left(.\right)}{\delta }$, marked in red, and the identity line in green. The systems are monostable except for column **(C)**, where it is bistable. The intersection points of the red line and the blue lines .+μ^*^ yield the locations of the extrema. Three different values for μ^*^ are shown: μ^*^_1_ = 1 (no burst, dark blue), μ^*^_2_ = 6 (medium-size burst, mid blue), μ^*^_3_ = 11 (strong burst, light blue). Through bursts, the modes are shifted toward smaller numbers of protein molecules. The second row shows the histograms of the protein distribution obtained from 5·10^4^ protein time-course simulations using the Gillespie algorithm. The distribution is shown for each burst size (same color as above). The location of the extrema corresponds well to the analytical results. The average values (dashed lines) match the deterministic steady state if *f* is linear, which is only the case in panel **(A)**. In **(C)**, large bursts generate a unimodal distribution (marked in light blue), although the system is bistable. In **(B,D)**, medium-size bursts lead to bimodality in spite of deterministic monostability (mid-blue line). Parameters are given in the Supplementary Table 1.

The number of fixed points thus depends on the structure of $\frac{\tilde{f}\left(.\right)}{\delta }$ and can be determined graphically as shown in the top row of Figure 1: The red line corresponds to the left hand-side of Equation (13) and has therefore the shape of $\tilde{f}$, and the identity line marked in green depicts the right hand-side. The steady states are located at the intersection points. Provided that the basal rate of protein production is nonzero, systems without feedback (panel A) or with non-cooperative positive feedback (panel B) can only have one stable fixed point. Those two cases are modeled by constant and by monotonically increasing, concave $\tilde{f}$, respectively. In case of cooperative feedback, which is characterized by a sigmoid structure of $\tilde{f}$, the system can be mono- or bistable (panels C and D).

### 3.3. Mathematical formulation using the chemical master equation

The master equation of the reaction system is obtained by inserting the rates and stoichiometry given in Equation (11) into Equation (3). It can be written as:

(14) $$\begin{array}{l} {\dot{p}}_{n}=\sum_{\mu \;=\;1}^{n}\left({\text{g}}_{\mu^{*}}(\mu )\frac{1}{\mu^{*}}f(n-\mu )p_{n-\mu }\right)-\frac{1}{\mu^{*}}f(n)p_{n}\\ \;+\delta (n+1)p_{n\;+\;1}-\delta np_{n}. \end{array}$$

${\text{g}}_{\mu^{*}}\left(\mu \right):=\frac{1}{\mu^{*}}\cdot {\left(1-\frac{1}{\mu^{*}}\right)}^{\mu -1}$, μ ∈ ℕ^+^, is the geometric probability mass function.

According to the calculation in Aquino et al. (2012) using Z-transform (a discrete version of the Laplace transform), the probability mass function in steady state (ṗ_*n*_ = 0∀*n*) can be formulated recursively as

(15) $$\begin{array}{l} \;p_{1}^{\text{ss}}=\frac{f(0)}{\delta \mu^{*}}p_{0}^{\text{ss}},\\ (n+1)p_{n\;+\;1}^{\text{ss}}=\frac{f(n)}{\delta \mu^{*}}p_{n}^{\text{ss}}+\frac{\mu^{*}-1}{\mu^{*}}np_{n}^{\text{ss}}. \end{array}$$

### 3.4. Calculation of central moments and modes

The ODEs for the expectation and the variance σ^2^ of the master equation read:

(16) $$\frac{d𝔼[N]}{dt}=𝔼[f(N)]-\delta 𝔼[N]$$

(17) $$\begin{array}{l} \frac{d\sigma^{2}(N)}{dt}=2\text{Cov}(N,f(N))-(𝔼[f(N)]-\delta 𝔼[N])\\ \;+2\mu^{*}𝔼[f(N)]-2\delta \sigma^{2}(N), \end{array}$$

where *N* is the discrete random variable of the number of protein molecules. The detailed calculation is shown in the Supplementary Material 2. In steady state, the central moments are therefore given by:

(18) $$𝔼[N]=\frac{𝔼[f(N)]}{\delta }$$

(19) $$\sigma^{2}(N)=\mu^{*}𝔼[N]+\frac{1}{\delta }\text{Cov}(N,f(N)).$$

Hence, the variance depends on the mean burst size μ^*^. For example, if *f* was constant (no feedback), σ^2^(*N*) = μ^*^𝔼[*N*] holds.

Let us now focus on the extrema of the probability distribution. In general, local maxima (modes) and minima obey the following conditions:

(20) $$p_{n-1}^{\text{ss}}\leq p_{n}^{\text{ss}},\;p_{n}^{\text{ss}}\geq p_{n\;+\;1}^{\text{ss}}\;\to \text{ maximum at }n$$

(21) $$p_{n-1}^{\text{ss}}\geq p_{n}^{\text{ss}},\;p_{n}^{\text{ss}}\leq p_{n\;+\;1}^{\text{ss}}\;\to \text{ minimum at }n,$$

if *n* > 0. Furthermore, one extremum necessarily occurs at *n* = 0.

Using Equation (15), one obtains the specific condition:

(22) $$p_{n\;+\;1}^{\text{ss}}-p_{n}^{\text{ss}}\;\begin{array}{c} \leq \\ \geq \end{array}\;0\;\Leftrightarrow \;\frac{f(n)}{\delta }\;\begin{array}{c} \leq \\ \geq \end{array}\;n+\mu^{*}.$$

Thus, the extrema satisfy the condition

(23) $$n=\left\lceil \frac{f(n)}{\delta }-\mu^{*}\right\rceil ,$$

where ⌈.⌉ denotes the ceiling function.

### 3.5. Comparison of the deterministic and stochastic descriptions

A comparison of the differential Equations (12) and (16) shows that the average (scaled by the volume) deviates from the deterministic variable if 𝔼[*f*(*N*)] ≠ *f*(𝔼[*N*]), which is usually the case when *f* is nonlinear. Inserting the Taylor series of *f*(*N*) around 𝔼[*N*], the ODE of the mean reads:

(24) $$\frac{d𝔼[N]}{dt}=f(𝔼[N])+\sum_{r\;=\;2}^{\infty }\left(\frac{1}{r!}z_{r}f^{\left(r\right)}(𝔼[N])\right)-\delta 𝔼[N]$$

with $z_{r}:=E\left[{\left(N-E\left[N\right]\right)}^{r}\right]$ denoting the *r*-th central moment of *N* and *f*^(*r*)^ being the *r*-th derivative of *f*. The mean of the CME is thus well described by the deterministic variable *c* only if *f* is almost linear or if higher central moments of *N* like the variance, skewness, kurtosis, etc. are small. As already mentioned, Equation (19) shows that bursting leads to a significant increase in the variance. Taken together, nonlinearity in the reaction can cause a deviation of *c*·*V* from 𝔼[*N*], which is expected to be enhanced through bursting.

Concerning the modes, a comparison of the conditions given in Equation (13) and in Equation (23) reveals a strong analogy if μ^*^ is small, so that stable fixed points can be associated with the maxima in the equilibrium probability mass function, and the unstable fixed points correspond to the minima. However, large bursts can disrupt this connection, as will be shown in the following section. The structure of *f* plays a minor role in this context.

#### 3.5.1. Large protein bursts can disrupt the connection between bistability and bimodality

In Figure 1, our previous calculations are visualized. Protein time-courses have been simulated using the Gillespie algorithm. For each plot, 5·10^4^ simulations were run and the histograms at a final time point *t*_*f*_ were plotted. In order to make sure that the steady state was approximately reached, several runs with random initial molecule numbers have been performed and compared to one another, and the simulated means and modes have been compared to the analytical values.

The first row of plots illustrates the analytical results, summarizing the findings from deterministic fixed point analysis as well as from the calculation of the stochastic extrema: According to Equation (13), the deterministic fixed points can be read from the intersection points of the graphs of $\frac{f\left(n\right)}{\delta }$ and *n*. The approximate location of the modes is given by the intersection of $\frac{f\left(n\right)}{\delta }$ and *n* + μ^*^, see Equation (23). From left to right, the structure of *f* was changed in order to check different feedback mechanisms. Furthermore, the mean burst size was varied in each case: Missing bursts ($\mu_{1}^{*}=1$), medium-size bursts ($\mu_{2}^{*}=6$) and large bursts ($\mu_{3}^{*}=11$) were considered. The plots below show the corresponding histograms of the simulations for the three different burst sizes. Moreover, the empirical mean of the distribution is highlighted.

First, let *f* ≡ *b* be constant. The simulations in panel (A) show that an increase in μ^*^ does not change the location of the empirical mean. However, the maximum of the distribution is biased toward smaller values, while the variance is enlarged. These observations are in perfect agreement to our calculations: The fixed point is located at $\frac{b}{\delta }$ and matches the empirical mean due to the linearity of *f*. The variance is given by $\sigma^{2}\left(N\right)=\mu^{*}\frac{b}{\delta }$, it therefore depends on the burst size. The mode fulfills the condition $n=\left\lceil \frac{b}{\delta }-\mu^{*}\right\rceil$ and is thus shifted to the left when μ^*^ is increased.

If *f* is a non-cooperative saturation curve (panel B), the deterministic steady state deviates from the empirical average of the distribution, and the bias is enlarged under bursting conditions, as stated before. Furthermore, the fixed point only matches with the maximum of the histogram when bursts are very small. Very interestingly, μ^*^ can even be large enough to generate a bimodal distribution which peaks at *n* = 0 and at a positive value. If the burst size is further increased, the distribution can eventually turn unimodal again, the only maximum being located at zero. This is also predicted by our analytical considerations, where the shift of the identity line by μ^*^ leads to the emergence of another intersection point with $\frac{f\left(n\right)}{\delta }$, corresponding to the formation of a minimum in the distribution, and a further shifting makes the intersection points vanish, so that the only maximum is found at *n* = 0. As a consequence, bursting can cause bimodality although the deterministic description predicts monostability.

Panel (C) addresses sigmoid functions *f*, which are often the result of protein oligomerizations leading to cooperative behavior. First, we have chosen the parameters such that the deterministic system is bistable. Again, an increase in μ^*^ shifts the modes to the left so that the deviation of the deterministic steady states increases. Under large bursts, as in the non-cooperative case, a unimodal distribution peaking at *n* = 0 can be observed. This is an example for a bistable system, which is unimodal.

By varying the parameters in the system with cooperative feedback, the results shown in panel (D) are obtained. The system is monostable, but it can get bimodal under bursting conditions. In contrast to the situation shown in (B), both maxima are located at positive molecule numbers.

All in all, large and rare bursts lead to an asymmetry in the protein production and degradation events, generating a skewed probability density with a large variance, that cannot be approximated by a normal distribution (cf. the association of deterministic and stochastic models via the Langevin equation in Gillespie, 2007). This disrupts the connection between deterministic fixed points and stochastic modes.

#### 3.5.2. Good agreement between the stochastic and deterministic descriptions in the thermodynamic limit

In spite of the preceding results, which reveals the possibility of huge deviations between the outcome of deterministic and stochastic models, the following calculation shows that in the theoretical thermodynamic limit *V* → ∞, *n* → ∞, s.t. $\frac{n}{V}$ is constant, a strong correlation between modes and fixed points exists.

Let us consider a system whose size is increased *s*-fold compared to system (Equation 11) (i.e., its volume is given by *s*·*V*). In order to maintain the concentrations, the rate of translation, which is formally a zeroth-order reaction, needs to be increased accordingly (it is thus given by $s\cdot \frac{1}{\mu^{*}}\tilde{f}$). In this case, the deterministic ODE remains unchanged, since Equation (12) is simply replaced by the identical formulation

(25) $$\dot{c}=\frac{s\tilde{f}(c)}{sV}-\delta c.$$

**Figure 2:**
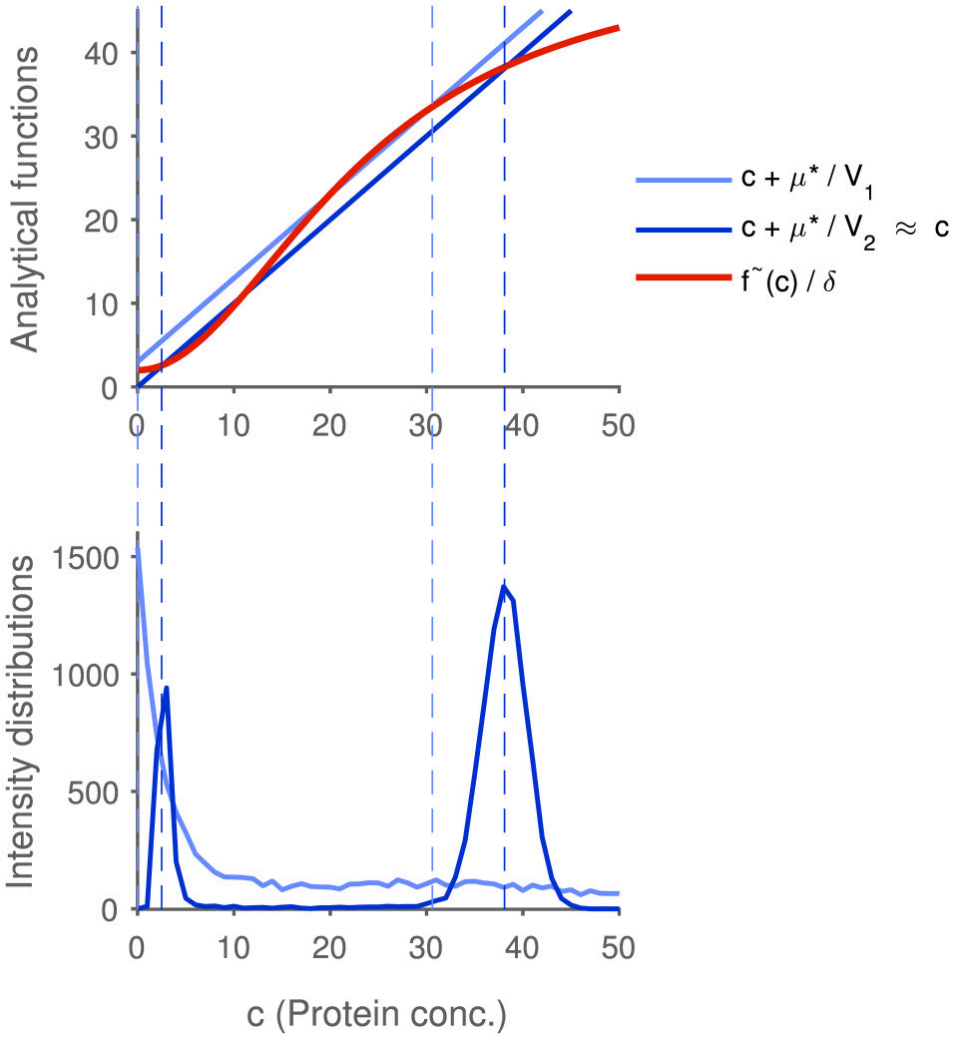
**Influence of the system size on the correspondence between deterministic and stochastic modeling results**. Two systems with differing sizes are compared: The volume *V*_1_ of system 1 (graphs in light blue) is chosen 50-fold smaller than the volume *V*_2_ of system 2 (graphs in dark blue), while the protein concentrations at the deterministic fixed points are identical. The intersections of the blue lines and the red line in the upper plot mark the analytical locations of the extrema in the protein probability mass function. The extrema of the larger system nearly coincide with the deterministic fixed points, since the expression $\frac{\mu *}{V_{2}}$ is almost negligible. The distributions in the bottom plot (obtained using the Gillespie algorithm) confirm these results: the larger system shows a clear bimodal distribution whose modes match the stable deterministic fixed points, while the modes of the small system are shifted, and the distribution is much broader. The dashed lines show that the analytical determination of the modes fits well to the simulations. Parameters are given in the Supplementary Table 2.

The condition for the stochastic modes reads

(26) $$n=csV=\left\lceil \frac{s\cdot \tilde{f}(c)}{\delta }-\mu^{*}\right\rceil$$

(27) $$\Rightarrow \;cV=\frac{\left\lceil \frac{s\cdot \tilde{f}(c)}{\delta }-\mu^{*}\right\rceil }{s}\;\overset{\;s\;\to \;\infty \;}{\to }\;\frac{\tilde{f}(c)}{\delta }$$

and thus matches the deterministic fixed point in the thermodynamic limit. The simulations shown in Figure 2, where protein distributions of two systems with differing sizes are compared, confirm this result. To put it in a slightly different way, the modes are in good agreement with the deterministic steady states if μ^*^ is small relative to the value of *n* at the extremum. However, note that from merely locating the deterministic fixed points in a bistable system, one cannot infer the average steady-state of the system, since the probability of a cell to be in one or the other state is unknown.

### 3.6. Feedback and burst characteristics influence the precision of the distribution and the robustness of bimodality

Next, we will give a qualitative estimate on the local precision (i.e., the inverse of the variance) of the probability distribution at the modes. The recursive formula (15) can be written as

(28) $$p_{n\;+\;1}^{\text{ss}}-p_{n}^{\text{ss}}=\frac{\frac{f(n)}{\delta }-(n+\mu^{*})}{\mu^{*}(n+1)}p_{n}^{\text{ss}}.$$

Therefore, the local change of the probability mass function relative to its height is large if

- $\left|\frac{f\left(n\right)}{\delta }-\left(n+\mu^{*}\right)\right|$ is large, while
- μ^*^ is small.

**Figure 3:**
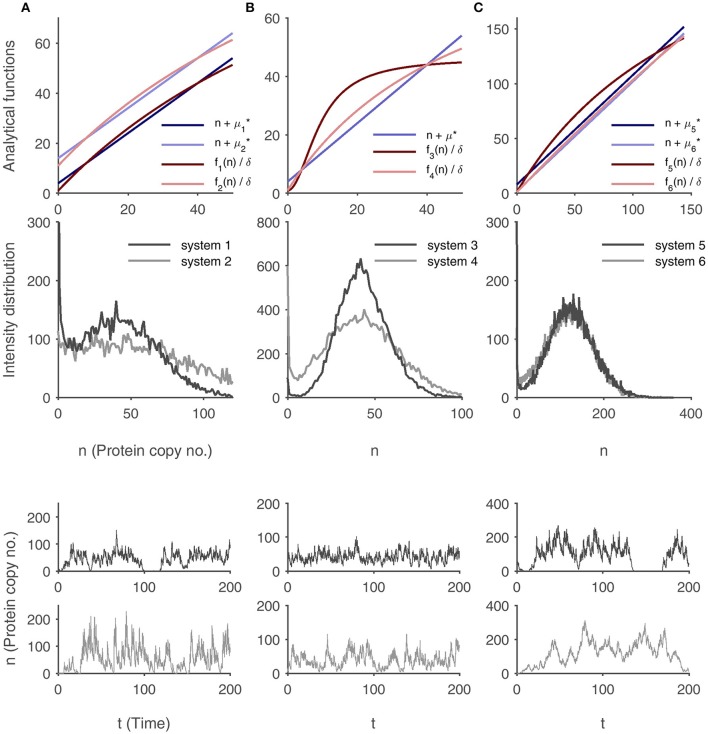
**Robustness of bimodality in different regulatory systems with feedback**. In each column, the robustness of the modes in two regulatory systems with varying burst sizes and varying functions *f* are compared based on the protein distributions and exemplary protein time-courses. **(A)** Comparison of two non-cooperative feedback regulations with differing burst sizes μ^*^_1_ < μ^*^_2_ and accordingly shifted functions *f*_1_ and *f*_2_ with identical shape. **(B)** Comparison of non-cooperative and cooperative regulation with identical burst size μ^*^. **(C)** Comparison of two non-cooperative regulations with identical basal protein expression and under differing burst sizes μ^*^_6_ < μ^*^_5_. In all cases, the system marked in dark colors (system 1, 3, and 5, respectively) exhibits a sharper distribution and a better separation of the modes is visible in the protein time-course simulations. Further explanations are given in the main text. Parameter values are listed in the Supplementary Table 3.

Under this condition, the local distribution forms sharp peaks around a maximum located at *n*. As a consequence, feedback structures and burst characteristics have a significant impact on the separation of the modes (without loss of generality, we do not include the effect of the degradation rate δ into our considerations). In the following, three scenarios will be portrayed which illustrate this result. They are visualized in Figure 3.

First, the burst size μ^*^ is varied, while $\frac{f\left(n\right)}{\delta }-\left(n+\mu^{*}\right)$ and the location of the modes are kept constant. This is most easily achieved by choosing two different burst sizes $\mu_{1}^{*}$ and $\mu_{2}^{*}$ with $\mu_{1}^{*}<\mu_{2}^{*}$ and by defining $f_{2}:=f_{1}+\delta \left(\mu_{2}^{*}-\mu_{1}^{*}\right)$ (i.e., the system with the larger average burst size has an enhanced basal protein production, while the shapes (derivatives) of *f*_1_ and *f*_2_ are identical). Then, $\frac{f_{1}\left(n\right)}{\delta }-\left(n+\mu_{1}^{*}\right)=\frac{f_{2}\left(n\right)}{\delta }-\left(n+\mu_{2}^{*}\right)$ holds true. The simulated histogram in Figure 3A shows that the system with the larger burst size does indeed have a broader distribution.

Next, the function *f* is modified while μ^*^ is held fixed. In this context, cooperative and non-cooperative regulation can be compared (cf. Figure 3B): Let $f_{3}\left(n\right):=b_{3}+v_{3}\frac{n^{h}}{n^{h}+K_{3}}$ with *h* > 1 describe Hill type kinetics (sigmoid function, cooperativity) and let $f_{4}\left(n\right):=b_{4}+v_{4}\frac{n}{n+K_{4}}$ be a Michaelis-Menten-type function (no cooperativity). Furthermore, to ensure comparability, let *b*_3_ = *b*_4_ (identical basal expression level), and let $\frac{f_{3}}{\delta }$ and $\frac{f_{4}}{\delta }$ have two identical intersection points with the line .+μ^*^. This guarantees that the modes of the probability distributions occur at the same protein molecule numbers. Then, it can be shown that for all *n*, $\left|\frac{f_{4}\left(n\right)}{\delta }-\left(n+\mu^{*}\right)|<|\frac{f_{3}\left(n\right)}{\delta }-\left(n+\mu^{*}\right)\right|$ holds (the proof is given in the Supplementary Material 3), so that the protein distribution of the cooperative system has sharper peaks.

Finally, both μ^*^ and $\frac{f\left(n\right)}{\delta }-\left(n+\mu^{*}\right)$ are varied. Let $f_{5}\left(n\right):=b_{5}+v_{5}\frac{n}{n+K_{5}}$, $f_{6}\left(n\right):=b_{6}+v_{6}\frac{n}{n+K_{6}}$, and let *b*_5_ = *b*_6_. Now, let $\mu_{5}^{*}>\mu_{6}^{*}$ and let $\frac{f_{5}\left(n\right)}{\delta }=n+\mu_{5}^{*}$ and $\frac{f_{6}\left(n\right)}{\delta }=n+\mu_{6}^{*}$ have an identical set of solutions. We are thus looking at two non-cooperative systems where the basal rate of protein production and the locations of the modes coincide, while the burst sizes and the curvatures of *f*_5_ and *f*_6_ differ. In this case, $\left|\frac{f_{5}\left(n\right)}{\delta }-\left(n+\mu_{5}^{*}\right)|>|\frac{f_{6}\left(n\right)}{\delta }-\left(n+\mu_{6}^{*}\right)\right|$ (cf. Supplementary Material 4), which counteracts the effect of the differing burst sizes. Explicit calculations are therefore required to determine which effect prevails. Interestingly, Figure 3C shows that the bimodality in the protein distribution of the system with larger bursts is even more precise.

Having addressed the probability mass function in steady state, single protein time-courses are now regarded. In a bimodal system, the robustness of the two stable steady states is crucial for its functionality: The protein level might fluctuate permanently between these states (small Mean first-passage times (MFPTs) of transitions between the inactive and active states, cf. van Kampen, 2007, Chapter 12), or it might tend to stay in one of the states with rare switching events (large MFPTs). The trajectories in Figure 3 show that a sharp bimodal distribution qualitatively correlates well with the robustness of the states. In Figure 3A, the fluctuations in the system with the lower burst level are much smaller, leading to more distinct switches between the modes. The protein level of the system with cooperative feedback in Figure 3B has small noise and stays in the active state, whereas the protein time-course in the non-cooperative circuit does not exhibit a clear separation of the modes. The time-courses in Figure 3C show that even systems with non-cooperative regulation are able to sustain two separate states, given that the nonlinearity of the feedback and the burst size are not too small, which severely contradicts the results of standard deterministic modeling.

## 4. Discussion

In this study, we have compared an ODE model based on the law of mass action with the corresponding CME formulation, implicitly stating that the master equation provides the much more realistic description of the biochemical reaction system. All deviations of the deterministic from the stochastic model have thus been interpreted as an indication of inadequacy of the ODE formalism. Indeed, as Gillespie states, “the stochastic approach is always valid whenever the deterministic approach is valid, and is sometimes valid when the deterministic approach is not” (Gillespie, 1976). One should still note that the CME, too, is based on several simplifying assumptions. Among these are the random, homogenous distribution of positions AND velocities of reactants, which is only a valid approximation when elastic molecular collisions predominate over reactive ones (Nicolis et al., 1974; Gillespie, 1992). Hence, we need to point out that although the CME approach often leads to experimentally verifiable results, this cannot be taken for granted. On the other hand, we can state that if significant mathematical deviations of the even more simplistic ODE approach from the CME model are observed, the deterministic description is almost surely unrealistic. Our study has led to the conclusion that although ODE modeling is quite a convenient and popular approach in many application fields, the use of deterministic models should be treated cautiously in the context of mesoscopic biochemical reaction systems.

The connection between deterministic and stochastic modeling has frequently been studied before. Several papers have reported on multi-component reaction systems that are monostable and bimodal, where bimodality is caused by the presence of components with very slow dynamics. These components can act as multi-level switches on fast downstream components (Qian et al., 2009; Ochab-Marcinek and Tabaka, 2010; Shu et al., 2011). Here, we have focused on nonlinear one-component reaction systems. A related study was previously conducted by Bishop and Qian (2010), where a phosphorylation-dephosphorylation cycle has been analyzed. They have shown that although the one-dimensional deterministic ODE model exhibits monostability, the weak nonlinearity in the reactions has the potential to cause stochastic bimodality, if the system size is sufficiently small. In their case, one of the stationary modes was invariably located at the zero state, whereas the other one was close to the deterministic steady state.

Here, we have systematically analyzed the effects of nonlinearity, but also of large stoichiometric coefficients in a flexible autoregulated gene expression system. In this context, we have proposed a graphical method which visualizes the impact of these system properties on the location of the modes and on their deviation from the deterministic fixed points. With the help of the graphics, it could be shown that monostable but bimodal systems can be constructed with both modes occurring at positive values, but only if the feedback is cooperative. We have seen that large stoichiometric coefficients can promote highly asymmetric, irregular fluctuation patterns in the copy numbers of the components. In our example, protein bursts allow for sudden and large increases in the number of protein molecules, whereas single degradation events reduce the number merely by one. Such instant jumps in molecule numbers have been explicitly excluded in the publications by Gillespie, where deterministic and stochastic variables were found to correspond well in sufficiently large systems (Gillespie, 2007, 2009). We have shown that when all reactions are linear, the mean and the deterministic variable coincide, but skewed fluctuations through large bursts lead to a shift of the mode away from the the mean. In the presence of nonlinear reaction propensities, the deterministic variable usually differs from the mean, and large bursts can even qualitatively change the modality of the distribution. One could argue that through a more detailed description of the bursting mechanism, large stoichiometric coefficients can to some extent be avoided. Nevertheless, there are components within a cell which usually occur at single-digit amounts (e.g., genes, mRNA), so that every reaction involving them is inevitably accompanied by a “large jump” relative to the molecule number. As a next step, the interplay of jumps, nonlinearities and reaction time-scales in a multi-component reaction system needs to be evaluated. Our preliminary results (not shown) indicate that those three factors together can further reduce the comparability of ODE and CME models.

This provokes the question of what kind of conclusions can still be drawn from deterministic modeling in small-scale reaction systems. In some biological contexts, stochasticity plays an important functional role: noise in certain signaling and gene regulation systems can lead to random transitions between different stable state and thus serve to create population heterogeneity, which makes cells more robust toward fluctuating environmental conditions. In this case, deterministic trajectories are certainly not realistic. But often, uniform cellular behavior can be observed. A coordinated hysteretic switch from one state to another, for example, is only possible if the modes are robustly separated. We have shown that although monostable systems can be bimodal with moderate switching frequency, a more robust bimodality is generated in a regime which is indeed deterministically bistable. In such cases, deterministic modeling might still provide valuable information on the dynamics of the system. For a more reliable description of biochemical processes in mesoscopic systems, however, we think that the use of stochastic modeling is virtually inevitable.

## Author contributions

AK supervised the project; AK and SH designed research; SH carried out analysis, simulations, and interpretation; All authors wrote and approved the manuscript.

### Conflict of interest statement

The authors declare that the research was conducted in the absence of any commercial or financial relationships that could be construed as a potential conflict of interest.

## References

[B1] Anderson D. F. (2008). Incorporating postleap checks in tau-leaping. J. Chem. Phys. 128:054103. 10.1063/1.2819665 18266441

[B2] Aquino T. Abranches E. Nunes A. (2012). Stochastic single-gene autoregulation. Phys. Rev. E Stat. Nonlin. Soft Matter Phys. 85(6 Pt 1):061913. 10.1103/PhysRevE.85.061913 23005133

[B3] Artyomov M. N. Das J. Kardar M. Chakraborty A. K. (2007). Purely stochastic binary decisions in cell signaling models without underlying deterministic bistabilities. Proc. Natl. Acad. Sci. U.S.A. 104, 18958–18963. 10.1073/pnas.0706110104 18025473 PMC2141890

[B4] Bishop L. M. Qian H. (2010). Stochastic bistability and bifurcation in a mesoscopic signaling system with autocatalytic kinase. Biophys. J. 98, 1–11. 10.1016/j.bpj.2009.09.055 20074511 PMC2800974

[B5] Chatterjee A. Vlachos D. G. Katsoulakis M. A. (2005). Binomial distribution based tau-leap accelerated stochastic simulation. J. Chem. Phys. 122:024112. 10.1063/1.1833357 15638577

[B6] Edelstein-Keshet L. (1988). Mathematical Models in Biology. New York, NY: McGraw-Hill.

[B7] Friedman N. Cai L. Xie X. S. (2006). Linking stochastic dynamics to population distribution: an analytical framework of gene expression. Phys. Rev. Lett. 97:168302. 10.1103/PhysRevLett.97.168302 17155441

[B8] Gallenberger M. zu Castell W. Hense B. A. Kuttler C. (2012). Dynamics of glucose and insulin concentration connected to the β-cell cycle: model development and analysis. Theor. Biol. Med. Model. 9:46. 10.1186/1742-4682-9-46 23164557 PMC3585463

[B9] Gillespie D. T. (1976). A general method for numerically simulating the stochastic time evolution of coupled chemical reactions. J. Comput. Phys. 22, 403–434. 10.1016/0021-9991(76)90041-3

[B10] Gillespie D. T. (1977). Exact stochastic simulation of coupled chemical reactions. J. Phys. Chem. 81, 2340–2361. 10.1021/j100540a008

[B11] Gillespie D. T. (1992). A rigorous derivation of the chemical master equation. Phys. A Stat. Mech. Appl. 188, 404–425. 10.1016/0378-4371(92)90283-V

[B12] Gillespie D. T. (2001). Approximate accelerated stochastic simulation of chemically reacting systems. J. Chem. Phys. 115:1716 10.1063/1.1378322 22239763

[B13] Gillespie D. T. (2007). Stochastic simulation of chemical kinetics. Annu. Rev. Phys. Chem. 58, 35–55. 10.1146/annurev.physchem.58.032806.104637 17037977

[B14] Gillespie D. T. (2009). Deterministic limit of stochastic chemical kinetics. J. Phys. Chem. B 113, 1640–1644. 10.1021/jp806431b 19159264 PMC2651820

[B15] Gunawardena J. (2014). Models in biology: ‘accurate descriptions of our pathetic thinking’. BMC Biol. 12:29. 10.1186/1741-7007-12-29 24886484 PMC4005397

[B16] Kremling A. Bettenbrock K. Gilles E. D. (2007). Analysis of global control of *Escherichia coli* carbohydrate uptake. BMC Syst. Biol. 1:42. 10.1186/1752-0509-1-42 17854493 PMC2148058

[B17] Kurtz T. G. (1972). The relationship between stochastic and deterministic models for chemical reactions. J. Chem. Phys. 57:2976.

[B18] Kurtz T. G. (1980). Biological Growth and Spread, volume 38 of Lecture Notes in Biomathematics. Berlin; Heidelberg: Springer.

[B19] McSweeney J. K. Popovic L. (2014). Stochastically-induced bistability in chemical reaction systems. Ann. Appl. Probab. 24, 1226–1268. 10.1214/13-AAP946

[B20] Nicolis G. Allen P. Van Nypelseer A. (1974). Some remarks on the theory of fluctuations around nonequilibrium states. Prog. Theor. Phys. 52, 1481–1497. 10.1143/PTP.52.1481

[B21] Ochab-Marcinek A. Tabaka M. (2010). Bimodal gene expression in noncooperative regulatory systems. Proc. Natl. Acad. Sci. U.S.A. 107, 22096–22101. 10.1073/pnas.1008965107 21135209 PMC3009792

[B22] Qian H. Shi P.-Z. Xing J. (2009). Stochastic bifurcation, slow fluctuations, and bistability as an origin of biochemical complexity. Phys. Chem. Chem. Phys. 11, 4861–4870. 10.1039/b900335p 19506761

[B23] Shinar G. Milo R. Martínez M. R. Alon U. (2007). Input output robustness in simple bacterial signaling systems. Proc. Natl. Acad. Sci. U.S.A. 104, 19931–19935. 10.1073/pnas.0706792104 18077424 PMC2148400

[B24] Shu C.-C. Chatterjee A. Dunny G. Hu W.-S. Ramkrishna D. (2011). Bistability versus bimodal distributions in gene regulatory processes from population balance. PLoS Comput. Biol. 7:e1002140. 10.1371/journal.pcbi.1002140 21901083 PMC3161895

[B25] To T.-L. Maheshri N. (2010). Noise can induce bimodality in positive transcriptional feedback loops without bistability. Science 327, 1142–1145. 10.1126/science.1178962 20185727

[B26] Tsimring L. S. (2014). Noise in biology. Rep. Prog. Phys. 77:026601. 10.1088/0034-4885/77/2/026601 24444693 PMC4033672

[B27] Tyson J. J. Othmer H. G. (1978). The dynamics of feedback control circuits in biochemical pathways, in Progress in Theoretical Biology, Vol. 5, eds Snell F. Rosen R. (New York, NY: Academic Press), 2–62.

[B28] van Kampen N. G. (2007). Stochastic Processes in Physics and Chemistry, 3rd Edn. Amsterdam: North Holland.

