# Supplementary material for: A Comparison of Deterministic and Stochastic Modeling Approaches for Biochemical Reaction Systems: On Fixed Points, Means, and Modes
Source: Front Genet. 2016 Aug 31;7:157. doi: 10.3389/fgene.2016.00157 (PMC5005346; doi:10.3389/fgene.2016.00157)
Supplement: Supplementary file 1 [file Presentation1.pdf]

Supplementary Material:
A Comparison of Deterministic and Stochastic Modeling Approaches for Biochemical Reaction Systems: On Fixed Points, Means, and Modes.

Sayuri K. Hahl* and Andreas Kremling
*Correspondence:
Sayuri Hahl
Sayuri.Hahl@tum.de

1 EXPECTATION OF THE GENERAL CHEMICAL MASTER EQUATION

Here, we derive Equation (8) from the main text. The state space can only take values in $(\mathbb{N}_0^+)^M$. By setting $\dot{p}_{\mathbf{n}} = 0$ for $\mathbf{n} \notin (\mathbb{N}_0^+)^M$, the dynamics of the expected value of the i-th component can be calculated as follows:

$$
\begin{aligned}
\dot{\mathbb{E}}[N_i] &= \sum_{\mathbf{n}\in\mathbb{Z}^M} n_i\,\dot{p}_{\mathbf{n}} \\
&= \sum_{\mathbf{n}\in\mathbb{Z}^M} \left(n_i \sum_{j=1}^{R} \left[\, w_j(\mathbf{n}-\mathbf{a}_j)\cdot p_{\mathbf{n}-\mathbf{a}_j} \;-\; w_j(\mathbf{n})\cdot p_{\mathbf{n}} \,\right] \right) \\
&= \sum_{j=1}^{R} \sum_{\mathbf{n}\in\mathbb{Z}^M} \left[\, (n_i + a_{ij})\cdot w_j(\mathbf{n})\cdot p_{\mathbf{n}} \;-\; n_i \cdot w_j(\mathbf{n})\cdot p_{\mathbf{n}} \,\right] \\
&= \sum_{j=1}^{R} \left(a_{ij} \cdot \mathbb{E}[w_j(\mathbf{N})] \right). \qquad (1)
\end{aligned}
$$

The third equality is obtained by index shifting.

2 MEAN AND VARIANCE OF THE CME OF THE PROTEIN EXPRESSION SYSTEM

Since the stoichiometric matrix of system (11) in the main text contains a random variable μ, we calculate the expectation of the CME analytically:

$$\begin{aligned}
\frac{d\,\mathbb{E}[N]}{dt} &= \sum_{n=0}^{\infty} n\,\dot{p}_n \\
&= \sum_{n=0}^{\infty}\left[\sum_{\mu=1}^{\infty}\left(\mathrm{g}_{\mu^*}(\mu)\,\frac{1}{\mu^*}\,f(n-\mu)\,n\,p_{n-\mu}\right) - \frac{1}{\mu^*}\,f(n)\,n\,p_n + \delta\,n\,(n+1)\,p_{n+1} - \delta\,n^2\,p_n\right] \\
&= \sum_{n=0}^{\infty}\left[\sum_{\mu=1}^{\infty}\left(\mathrm{g}_{\mu^*}(\mu)\,\frac{1}{\mu^*}\,f(n)\,(n+\mu)\,p_n\right) - \frac{1}{\mu^*}\,f(n)\,n\,p_n + \delta\,(n-1)\,n\,p_n - \delta\,n^2\,p_n\right] \\
&= \sum_{n=0}^{\infty}\left[\left(\sum_{\mu=1}^{\infty}\mu\,\mathrm{g}_{\mu^*}(\mu)\right)\frac{1}{\mu^*}\,f(n)\,p_n + \left(\sum_{\mu=1}^{\infty}\mathrm{g}_{\mu^*}(\mu)\right)\frac{1}{\mu^*}\,f(n)\,n\,p_n - \frac{1}{\mu^*}\,f(n)\,n\,p_n - \delta\,n\,p_n\right] \\
&= \sum_{n=0}^{\infty}\left[\mu^*\,\frac{1}{\mu^*}\,f(n)\,p_n - \delta\,n\,p_n\right] = \mathbb{E}[f(N)] - \delta\,\mathbb{E}[N]
\end{aligned} \tag{2}$$

The third equality is obtained by index shifting in n (note that $p_n = 0$ for negative n). In the fifth row, the fact that $\mathrm{g}_{\mu^*}(\mu)$ is a probability distribution with mean μ^* was used.

Similar to the calculation of the mean, the variance σ^2 of N is computed. First of all, the ODE of the second moment $\mathbb{E}[N^2]$ is given by

$$\begin{aligned}
\frac{d\,\mathbb{E}[N^2]}{dt} &= \sum_{n=0}^{\infty} n^2\,\dot{p}_n \\
&= \sum_{n=0}^{\infty}\left[\sum_{\mu=1}^{\infty}\left(\mathrm{g}_{\mu^*}(\mu)\,\frac{1}{\mu^*}\,f(n-\mu)\,n^2\,p_{n-\mu}\right) - \frac{1}{\mu^*}\,f(n)\,n^2\,p_n + \delta\,n^2\,(n+1)\,p_{n+1} - \delta\,n^3\,p_n\right] \\
&= \sum_{n=0}^{\infty}\left[\sum_{\mu=1}^{\infty}\left(\mathrm{g}_{\mu^*}(\mu)\,\frac{1}{\mu^*}\,f(n)\,(n+\mu)^2\,p_n\right) - \frac{1}{\mu^*}\,f(n)\,n^2\,p_n + \delta\,(n-1)^2\,n\,p_n - \delta\,n^3\,p_n\right] \\
&= \sum_{n=0}^{\infty}\Bigg[\left(\sum_{\mu=1}^{\infty}\mu^2\,\mathrm{g}_{\mu^*}(\mu)\right)\frac{1}{\mu^*}\,f(n)\,p_n + 2\left(\sum_{\mu=1}^{\infty}\mu\,\mathrm{g}_{\mu^*}(\mu)\right)\frac{1}{\mu^*}\,f(n)\,n\,p_n + \\
&\qquad + \left(\sum_{\mu=1}^{\infty}\mathrm{g}_{\mu^*}(\mu)\right)\frac{1}{\mu^*}\,f(n)\,n^2\,p_n - \frac{1}{\mu^*}\,f(n)\,n^2\,p_n - 2\,\delta\,n^2\,p_n + \delta\,n\,p_n\Bigg] \\
&= \sum_{n=0}^{\infty}\left[(2\,\mu^*-1)\,f(n)\,p_n + 2\,f(n)\,n\,p_n - 2\,\delta\,n^2\,p_n + \delta\,n\,p_n\right] \\
&= (2\,\mu^*-1)\,\mathbb{E}[f(N)] + 2\,\mathbb{E}[N\,f(N)] - 2\,\delta\,\mathbb{E}[N^2] + \delta\,\mathbb{E}[N]
\end{aligned} \tag{3}$$

The ordinary differential equation of the variance $\sigma^2 = \mathbb{E}[N^2] - \mathbb{E}[N]^2$ reads:

$$\begin{aligned} \frac{d\sigma^2(N)}{dt} &= \frac{d\,\mathbb{E}[N^2]}{dt} \;-\; 2\,\mathbb{E}[N]\,\frac{d\,\mathbb{E}[N]}{dt} \\ &= 2\,\mathrm{Cov}(N, f(N)) \;-\; (\mathbb{E}[f(N)] \;-\; \delta\,\mathbb{E}[N]) \;+\; 2\,\mu^*\,\mathbb{E}[f(N)] \;-\; 2\,\delta\,\sigma^2(N) \end{aligned} \tag{4}$$

3 COMPARISON OF COOPERATIVE AND NON-COOPERATIVE SYSTEMS

Let

$$f_3(n) = b + v_3 \frac{n^h}{n^h + K_3}, \quad h \geq 2, \quad \text{and} \tag{5}$$

$$f_4(n) = b + v_4 \frac{n}{n + K_4} \tag{6}$$

be two Hill-type functions. Moreover, let $\frac{f_3}{\delta}$ and $(. + \mu^*)$ possess two positive intersection points n_{min} and n_{max}, which are identical to the only two positive intersection points of $\frac{f_4}{\delta}$ and $(. + \mu^*)$. In this section, we show that

$$\left| \frac{f_3(n)}{\delta} - (n + \mu^*) \right| \;\geq\; \left| \frac{f_4(n)}{\delta} - (n + \mu^*) \right| \tag{7}$$

holds for all $n \geq 0$.

The proof is given as follows: Since f_4 is concave, for the above conditions to be met, it can easily be derived that

- $\frac{f_4(n)}{\delta} < n + \mu^* \quad \forall n \in [0, n_{min})$
- $\frac{f_4(n)}{\delta} > n + \mu^* \quad \forall n \in (n_{min}, n_{max})$
- $\frac{f_4(n)}{\delta} < n + \mu^* \quad \forall n \in (n_{max}, \infty)$.

Therefore, it suffices to show that

- $f_3(n) < f_4(n) \quad \forall n \in (0, n_{min})$
- $f_3(n) > f_4(n) \quad \forall n \in (n_{min}, n_{max})$
- $f_3(n) < f_4(n) \quad \forall n \in (n_{max}, \infty)$.

As a first step, we prove that $v_3 < v_4$. Solving both conditions $f_3(n_{min}) = f_4(n_{min})$ and $f_3(n_{max}) = f_4(n_{max})$ for K_3 and equating the expressions leads to

$$\frac{v_3}{v_4} = \frac{n_{max}^h - n_{min}^h}{n_{max}^h - n_{min}^h + K_4(n_{max}^{h-1} - n_{min}^{h-1})}. \tag{8}$$

This fraction is smaller than 1.

First, we use this fact to prove that 0, n_{min} and n_{max} are the only intersection points of f_3 and f_4. Reformulating the equation $f_3(n) = f_4(n)$ leads to the following conditions for nonzero solutions:

$$g(n) := n^{h-1}\left[(v_4 - v_3)\,n \;-\; v_3\,K_4\right] \;+\; v_4\,K_3 \;=\; 0 \tag{9}$$

The extreme values of g fulfill the condition

$$g'(n) := n^{h-2}\,[h\,(v_4 - v_3)\,n \;-\; (h-1)\,v_3\,K_4] \;=\; 0 \tag{10}$$

which has at most one positive solution. From Rolle's theorem, one can conclude that g has at most two positive roots. Hence, the only intersections in positive space occur at n_{min} and n_{max}.

Due to the fact that for $n \to \infty$, f_3 and f_4 converge to $b + v_3$ and $b + v_4$, respectively, the relation $v_4 > v_3$ and the fact that $f_3(n) \neq f_4(n)$ for all $n > n_{max}$ shows that $f_4(n) > f_3(n)$ necessarily holds for all $n \in (n_{max}, \infty)$.

On the other hand, as $f_3'(0) = 0 < \frac{v_4}{K_4} = f_4'(0)$, the inequality $f_3(n) < f_4(n)$ is fulfilled in the interval $(0, n_{min})$.

Finally, it remains to prove that $f_4 < f_3$ in (n_{min}, n_{max}). It is sufficient to show that $f_3'(n_{min}) \neq f_4'(n_{min})$ or $f_3'(n_{max}) \neq f_4'(n_{max})$, so that f_3 and f_4 truly intersect. Suppose that

$$v_3\frac{K_3\,h\,n_{min}^{h-1}}{(n_{min}^h + K_3)^2} = f_3'(n_{min}) = f_4'(n_{min}) = v_4\frac{K_4}{(n_{min} + K_4)^2}. \tag{11}$$

Then, by exploiting the relation $f_3(n_{min}) = f_4(n_{min})$, this equation can be reformulated as

$$\frac{K_3\,v_4\,h}{K_4\,v_3} = n_{min}^{h-1}. \tag{12}$$

In an analogous manner, the assumption $f_3'(n_{max}) = f_4'(n_{max})$ leads to the contradictory equation

$$\frac{K_3\,v_4\,h}{K_4\,v_3} = n_{max}^{h-1}, \tag{13}$$

which is why the original assumption was wrong and $f_3'(n_{min}) \neq f_4'(n_{min})$ or $f_3'(n_{max}) \neq f_4'(n_{max})$ holds true. □

4 COMPARISON OF COOPERATIVE SYSTEMS WITH DIFFERING BURST SIZES

Let

$$f_5(n) = b + v_5\frac{n}{n + K_5} \quad \text{and} \tag{14}$$

$$f_6(n) = b + v_6\frac{n}{n + K_6} \tag{15}$$

be two functions describing Michaelis-Menten type kinetics. Let $\mu_5^* > \mu_6^*$ and let $\frac{f_5(n)}{\delta} = n + \mu_5^*$ and $\frac{f_6(n)}{\delta} = n + \mu_6^*$ have an identical set of solutions $\{n_{min}, n_{max}\}$. Our aim is to show that

$$\left|\frac{f_5(n)}{\delta} - (n + \mu_5^*)\right| > \left|\frac{f_6(n)}{\delta} - (n + \mu_6^*)\right| \tag{16}$$

holds true.

First, note that for $l \in \{5, 6\}$,

- $\frac{f_l(n)}{\delta} < n + \mu_l^* \quad \forall n \in [0, n_{min})$
- $\frac{f_l(n)}{\delta} > n + \mu_l^* \quad \forall n \in (n_{min}, n_{max})$
- $\frac{f_l(n)}{\delta} < n + \mu_l^* \quad \forall n \in (n_{max}, \infty)$.

Therefore, it suffices to show that

- $\frac{f_6(n)}{\delta} - \mu_6^* > \frac{f_5(n)}{\delta} - \mu_5^* \quad \forall n \in (0, n_{min})$
- $\frac{f_6(n)}{\delta} - \mu_6^* < \frac{f_5(n)}{\delta} - \mu_5^* \quad \forall n \in (n_{min}, n_{max})$
- $\frac{f_5(n)}{\delta} - \mu_6^* > \frac{f_5(n)}{\delta} - \mu_5^* \quad \forall n \in (n_{max}, \infty)$.

K_5, K_6, v_5, and v_6 can be determined analytically, using the relations above. The results are:

$$K_5 = \frac{n_{min}\, n_{max}}{\mu_5^* - \frac{b}{\delta}}, \tag{17}$$

$$K_6 = \frac{n_{min}\, n_{max}}{\mu_6^* - \frac{b}{\delta}}, \tag{18}$$

$$v_5 = \frac{\delta\,(n_{min} + \mu_5^* - \frac{b}{\delta})\,(n_{max} + \mu_5^* - \frac{b}{\delta})}{\mu_5^* - \frac{b}{\delta}}, \tag{19}$$

$$v_6 = \frac{\delta\,(n_{min} + \mu_6^* - \frac{b}{\delta})\,(n_{max} + \mu_6^* - \frac{b}{\delta})}{\mu_6^* - \frac{b}{\delta}} \tag{20}$$

The evaluation of the first derivative of $\frac{f_5}{\delta}$ and $\frac{f_6}{\delta}$ at the intersection points reads

$$\frac{d}{dn}\left(\frac{f_l(n)}{\delta} + \mu_l^*\right)\Big|_{n=n_{min}} = \frac{f_l'(n_{min})}{\delta} = \frac{n_{max}\, n_{min} + n_{max}\,(\mu_l^* - \frac{b}{\delta})}{n_{max}\, n_{min} + n_{min}\,(\mu_l^* - \frac{b}{\delta})}, \tag{21}$$

$$\frac{d}{dn}\left(\frac{f_l(n)}{\delta} + \mu_l^*\right)\Big|_{n=n_{max}} = \frac{f_l'(n_{max})}{\delta} = \frac{n_{max}\, n_{min} + n_{min}\,(\mu_l^* - \frac{b}{\delta})}{n_{max}\, n_{min} + n_{max}\,(\mu_l^* - \frac{b}{\delta})} \tag{22}$$

for $l \in \{5, 6\}$. The dependence of the expressions on μ_l^* shows that

$$\frac{f_5'(n_{min})}{\delta} > \frac{f_6'(n_{min})}{\delta} \quad \text{and} \tag{23}$$

$$\frac{f_5'(n_{max})}{\delta} < \frac{f_6'(n_{max})}{\delta}. \tag{24}$$

The equation $\frac{f_6'(n)}{\delta} - \frac{f_5'(n)}{\delta} = 0$ has only one positive solution (analytical calculation not shown), so that n_{min} and n_{max} are the only intersection points of $\frac{f_5(n)}{\delta} - n - \mu_5^*$ and $\frac{f_6(n)}{\delta} - n - \mu_6^*$ according to Rolle's theorem. Hence, one can conclude from the derivatives at the intersection points that the above conditions are fulfilled and that the proof is complete. □

5 PARAMETER VALUES USED IN SIMULATIONS

The histograms in Figures 1, 2, and 3 show the distributions of $5 \cdot 10^4$, $1 \cdot 10^4$, and $2 \cdot 10^4$ protein simulations at a final time t_f, respectively. The parameters are listed below.

Supplementary Table 1. Functions and parameters used in the simulations shown in Figure 1.

(A)	(B)	(C)	(D)
$f(x) = b$	$f(x) = b + v\frac{x}{K+x}$	$f(x) = b + v\frac{x^2}{K+x^2}$	$f(x) = b + v\frac{x^2}{K+x^2}$
$\delta = 1$	$\delta = 1$	$\delta = 1$	$\delta = 1$
$b = 10$	$b = 2$	$b = 2$	$b = 7$
	$v = 61.75$	$v = 58.56$	$v = 41.25$
	$K = 25$	$K = 550$	$K = 400$
$\mu_1^* = 1$	$\mu_1^* = 1$	$\mu_1^* = 1$	$\mu_1^* = 1$
$\mu_2^* = 6$	$\mu_2^* = 6$	$\mu_2^* = 6$	$\mu_2^* = 6$
$\mu_3^* = 11$	$\mu_3^* = 11$	$\mu_3^* = 11$	$\mu_3^* = 11$

Supplementary Table 2. Functions and parameters used in the simulations shown in Figure 2.

$f(x) = b + v\frac{x^2}{K+x^2}$
$\delta = 1$
$b = 2$
$v = 50$
$K = 550$
$\mu^* = 3$
$V_1 = 1$
$V_2 = 50$

Supplementary Table 3. Functions and parameters used in the simulations shown in Figure 3.

(A)	(B)	(C)
$f_1(x) = b + v_1 \frac{x}{K_1+x}$	$f_3(x) = b + v_3 \frac{x^2}{K_3+x^2}$	$f_5(x) = b + v_5 \frac{x}{K_5+x}$
$f_2(x) = b + v_2 \frac{x}{K_2+x}$	$f_4(x) = b + v_4 \frac{x}{K_4+x}$	$f_6(x) = b + v_6 \frac{x}{K_6+x}$
$\delta = 1$	$\delta = 1$	$\delta = 1$
$b = 1$	$b = 1$	$b = 1$
$S_{min} = 8$	$S_{min} = 4$	$S_{min} = 30$
$S_{max} = 40$	$S_{max} = 40$	$S_{max} = 120$
$\mu_1^* = 4$	$\mu^* = 4$	$\mu_5^* = 8$
$\mu_2^* = 14$		$\mu_6^* = 2$
